# Reliability and validity of the Shoulder Pain and Disability Index in a sample of patients with frozen shoulder

**DOI:** 10.1186/s12891-023-06268-2

**Published:** 2023-03-22

**Authors:** Davide Venturin, Gabriele Giannotta, Leonardo Pellicciari, Alex Rossi, Denis Pennella, Michela Goffredo, Antonio Poser

**Affiliations:** 1 Kinè Physiotherapic and Orthopedic Center, San Vendemiano, Treviso, Italy; 2 Department of Medicine and Health Science “Vincenzo Tiberio”, University of Molise, Campobasso, Italy; 3 In Corpore Sano, Physiotherapic Clinic, Lecce, Italy; 4 IRCCS Istituto Delle Scienze Neurologiche Di Bologna, Bologna, Italy; 5 University of Rome “Tor Vergata”, Rome, Italy; 6 “Manual Therapy Lab” Clinic, Bari, Italy; 7 Department of Neurological and Rehabilitation Sciences, IRCCS San Raffaele Roma, Via Della Pisana 235, 00163 Rome, Italy; 8 University of Padua, Padua, Italy

**Keywords:** Outcome Assessment, Health Care, Psychometrics, Reproducibility of Results, Frozen Shoulder, SPADI

## Abstract

**Background:**

The Shoulder and Pain Disability Index (SPADI) is a widely used outcome measure. The aim of this study is to explore the reliability and validity of SPADI in a sample of patients with idiopathic frozen shoulder.

**Methods:**

The SPADI was administered to 124 patients with idiopathic frozen shoulder. A sub-group of 29 patients were retested after 7 days. SPADI scores were correlated with other outcome measures (i.e., Disabilities of the Arm, Shoulder and Hand Questionnaire – DASH; Numerical Pain Rating Scale—NPRS; and 36-item Short Form Health Survey—SF-36) to examine construct validity. Structural validity was assessed by a Two-Factors Confirmatory Factor Analysis (CFA). Internal consistency, test–retest reliability, and measurement error were also analyzed.

**Results:**

The construct validity was satisfactory as seven out of eight of the expected correlations formulated (≥ 75%) for the subscales were satisfied. The CFA showed good values of all indicators for both Pain and Disability subscales (Comparative Fit Index = 0.999; Tucker-Lewis Index = 0.997; Root Mean Square Error of Approximation = 0.030). Internal consistency was good for pain (α = 0.859) and disability (α = 0.895) subscales. High test–retest reliability (Intraclass correlation coefficient [ICC]) was found for pain (ICC = 0.989 [95% Confidence Interval (CI = 0.975–0.995]) and disability (ICC = 0.990 [95% CI = 0.988–0.998]). Standard Error of Measurement values of 2.27 and 2.32 and Minimal Detectable Change values of 6.27 and 6.25 were calculated for pain and disability subscales, respectively.

**Conclusion:**

The SPADI demonstrated satisfactory reliability and validity properties in a sample of patients with idiopathic frozen shoulder.

## Background

Idiopathic Frozen Shoulder (FS) is often characterized by severe shoulder pain and functional restrictions of both active and passive shoulder motion [1]. These restrictions are usually unremarkable in radiographs of the glenohumeral joint, and there is no gold standard diagnostic test for this syndrome [2]. Based on the clinical findings, an appropriate diagnostic process should include: limitations of both active and passive Range of Motion (ROM) in different planes of movement, especially in external rotation at varying degrees of shoulder abduction [3], and a normal shoulder radiograph in order to exclude/eliminate any additional pathologies that may exist [4].

Delays in the clinical diagnostic process incomplete or unclear prognostic information, and lack of a standard rehabilitation program are issues that may increase the patients’ frustration and the socio-cultural impact that FS has on their life [5]. FS generally impacts multiple aspects of daily life, from leisure time to work: everything is modified to adapt to the related disabilities [6].

In order to achieve an effective therapeutic alliance, it is essential to use clear and valid information that is based on the best available evidence. This will allow a tailored rehabilitation program to be developed based on the patient's needs and fears. In this context, health indicators could be used to overcome the discrepancy between clinicians’ and patients' perceptions [7] of disabling conditions. To this extent, the Patient Reported Outcome Measures system (PROMs) [8], which measures patients’ perceptions of their health status, clinical outcomes, mobility, and quality of life [9], can systematically assess the patients’ point of view, leading to improved communication and patient management using a personalized care approach [10].

Based on our literature review, only one study has assessed the responsiveness of the Shoulder Pain and Disability Index (SPADI) in a sample of patients with FS, which demonstrated that it could be an appropriate PROMs to assess the point of view of this population [11]. This self-administered index consists of 13 items divided into two subscales: 5 items for pain and 8 items for disability [7], which are also the two aspects that most concern patients with FS [9]. The SPADI is a practical outcome measure that can be completed by patients in less than 5 min and is easily scored by clinicians [12]. SPADI has been shown to have excellent reliability and construct validity in the assessment of shoulder impairments [13], mostly in patients presenting at the primary care level with shoulder pain [14, 15]. Moreover, SPADI is one of the most commonly used PROMs to assess pain and disability in patients with FS [16], even though it has not undergone any specific scientific validation in populations with FS. Therefore, the aim of this study was to examine the reliability and validity of SPADI in a sample of Italian patients with FS.

## Methods

### Inclusion and exclusion criteria

Patients were recruited through convenience sampling in two Italian private physical therapy clinics, over a 3-year period between 2019 and 2021 if they met the following inclusion criteria: 1) over 18 years of age; 2) clinical diagnosis of idiopathic FS by orthopedic surgeons and physiotherapists based on the following findings: *a)* gradual and progressive onset of shoulder pain; *b)* a restriction of both glenohumeral active and passive ROM in multiple planes; *c)* sleep-disturbing night pain and/ or firm end-feel at the end ranges of movements, especially in external rotation at variable degrees of shoulder abduction that occurs for at least 1 month [3]; and 3) normal shoulder radiograph [4]. Patients were excluded if they presented with red flags (e.g., neoplasia, fractures or glenohumeral dislocation) [17], secondary stiff shoulder (following the ISAKOS 2015 classification) [2], history of trauma [2], positive history of cognitive impairments, or inability to understand the Italian language.

All procedures involving human participants were in accordance with the Helsinki declaration and its later amendments. Personal information and informed consent were obtained from all patients to participate in this study.

### Outcome measures

#### Shoulder and Pain Disability Index (SPADI)

The Italian version of the SPADI used in this study was cross-culturally adapted and validated by Marchese C, et al., available elsewhere [18]. The SPADI has already been used to assess shoulder dysfunction in patients with neck cancer [18], in patients after shoulder surgery for anterior instability [19], and in patients with non-specific shoulder pain [20].

Before the compilation process, patients were instructed to place a mark on the Visual Analogue Scale (VAS) for each of the 13 items [18]. The results of each subscale were added up and converted into a score out of 100: the closer the score is to 100, the greater the pain and disability [18]. Patients could only mark one item in each subscale as “not applicable” and, in this case, the item was omitted from the total score. If a patient marked more than two items as “non-applicable”, no score was calculated [21]. Previous studies have reported an MDC of 17 points using the SPADI in patients with FS [11] and an MDC of 18 points using the German SPADI in patients post shoulder arthroplasty [21].

#### Disability of Arm, Shoulder and Hand Questionnaire (DASH)

The DASH is an outcome measure that assesses the ability of an upper extremity to perform activities of daily living regardless of the site(s) and nature of musculoskeletal pathology [22]. The DASH questionnaire is composed of 30 items assessing disability and symptoms. Items on the DASH test the degree of difficulty in performing various physical, social, and work-related daily activities together with the impact on the sleep routine and the patient’s perception of him/considering the upper extremity problems [23]. For each question, patients rate their difficulty on a five-point Likert scale ranging from ‘‘no difficulty or no symptoms’’ (scores 1) to ‘‘unable to perform activity or severe symptoms’’ (scores 5) [24]. The DASH score ranges from 0 (no disability) to 100 (severest disability) and cannot be calculated if there are more than 3 missing items [23]. In this study, the cross culturally adapted and validated Italian version was used [25].

#### Numerical Pain Rating Scale (NPRS)

The NPRS is an eleven-point measure of pain: the patient rates pain from 0 (no pain) to 10 (worst imaginable pain) [26]. The NPRS has shown good responsiveness in shoulder pain [27, 28] and, in this study, it was used to assess the patient's pain perception in the previous last week.

#### 36-Item Short Form Health Survey questionnaire (SF-36)

The SF-36 is a tool that assesses Health-Related Quality of Life [29]. The SF-36 measures eight domains: Physical Functioning (PF), Role Physical (RP), Bodily Pain (BP), General Health (GH), Vitality (VT), Social Functioning (SF), Role Emotional (RE), and Mental Health (MH) [30]. The SF- 36 has shown good properties in assessing patients with different shoulder disorders [31]. The validated Italian version was used in this study [32].

### Procedures

The SPADI was administered to each patient together with the DASH, NPRS, and SF-36. Clinical and demographic data were also acquired during the recruitment phase (i.e., age, gender) through an interview conducted by a physiotherapist. Each questionnaire was self-reported by the patients.

### Statistical analysis

This study has followed the definitions and procedures proposed by the COSMIN initiative [33] in the process of evaluating the psychometric properties of SPADI.

Structural validity was assessed by a two-factors Confirmatory Factor Analysis (CFA) [34] using the following three indicators [35]: 1) Comparative Fit Index (CFI) and the Tucker-Lewis Index (TLI) ≥ 0.95, 2) Root Mean Square Error of Approximation (RMSEA) ≤ 0.06, and 3) Standardized Root Mean Square Residual (SRMR) ≤ 0.08.

Reliability was assessed as internal consistency, test–retest reliability, and measurement error. These analyses were performed on both subscales (Pain and Disability) separately. Internal consistency was evaluated according to these criteria: 1) Cronbach’s Alpha (α) [36], where values are recommended between 0.70 and 0.95. [37], 2) α if an item was deleted, where the alpha was calculated after removing each item in turn; values below the total Cronbach’s alpha are expected [38], and 3) items to total correlation, which are the nonparametric correlations (based on Spearman’s ϱ) between each item and its rest score (i.e. the total score minus the item score); values ≥ 0.40 were considered satisfactory [39].

To examine test–retest reliability, the Intraclass Correlation Coefficient (ICC_2,1_) with 95% Confidence Intervals (CI) [40] was calculated. The minimal ICC value required for a reliable measure of groups is > 0.75 [41], but values > 0.90 are considered essential to achieve excellent reliability in a clinical measurement at the individual level [42]. The Standard Error of Measurement (SEM) and the Minimum Detectable Change (MDC) were used to test the measurement error [43]. The SEM was calculated with the following formula: SD*√(1–ICC), where SD is the pooled standard deviation of the measurements, and the ICC value is the test–retest reliability. The MDC was calculated by multiplying the SEM by 1.96, which is the z-score associated with the 95% confidence level and the square root of 2.

The outcome measures were used for a comparative analysis to study the validity of two constructs: for pain, the SPADI Pain subscale score was correlated with the NPRS; for disability, the SPADI Disability subscale score was correlated with the DASH score even though the DASH measures disability of the upper limb and is not specific to the shoulder [44].

Construct validity was examined through an *a-priori* hypothesis test using the magnitudes of the Spearman’s rank correlation (r_s_) coefficients between the SPADI and the other three scales (DASH, NPRS, and SF-36).The cut-offs of r_s_ > 0.60, 0.30 < r_s_ < 0.60, and r_s_ < 0.30 represent strong, moderate, and weak correlations, respectively [45]. Construct validity was considered satisfactory (≥ 75%), moderate (< 75%, and ≥ 50%), or low (< 50%) depending on the percentage of expected hypotheses fulfilled [46].

Based on the expected correlations, an *a-priori* hypotheses testing was conducted for the following assumptions:

- For the Pain subscale:

1. the positive correlation between the Pain subscale and the NPRS scores is > 0.60 because they measure the same construct, and this correlation value was found in another SPADI validation study [47];
2. the positive correlation between the Pain subscale and the DASH total scores is > 0.60 as found in other SPADI validation studies [48, 49];
3. the negative correlation between the Pain subscale and the Physical Functioning (PF) subscale of the SF-36 is > 0.30 since shoulder pain globally compromises the physical health status [50];
4. the negative correlation between the Pain subscale and the Bodily Pain (BP) subscale of the SF-36 scores is > 0.30 and < 0.60, based on their similar constructs.

- For the Disability subscale:

1. the positive correlation between the Disability subscale and the DASH total scores is > 0.60, as reported in other SPADI validation studies [19, 48, 49];
2. the positive correlation between the Disability subscale and the NPRS scores is between > 0.30 and < 0.60, since pain showed a moderate correlation with disability in similar patients [15, 47];
3. the negative correlation between the Disability subscale and the Role Physical (RP) subscale of the SF-36 scores is > 0.30 [47, 51];
4. the negative correlation between the Disability subscale and the Social Functioning (SF) subscale of the SF-36 scores is > 0.30 [47, 51].

The R-package lavaan was used to run the CFA, while the SPSS package, version 21 for Windows (SPSS Inc., Chicago, IL; 2004) was used for all other statistical analyses. Statistical significance was set as *p* < 0.05 for all analyses.

## Results

### Subjects

One hundred and twenty-four (mean ± SD age = 55.2 ± 7.7 years, 46.8% male) subjects with FS were included in this study. Detailed demographic and clinical characteristics are presented in Table 1. A sub-group of twenty-nine patients were used to conduct the test–retest after seven days.

**Table 1:** Demographic and clinical characteristics of the sample (*N* = 124)

Variable	Mean ± SD	Frequency (%)
Age, *years*	55.2 ± 7.7	
Gender		
Male		58 (46.8%)
Female		66 (53.2%)
Affected shoulder		
Right		55 (44.4%)
Left		69 (55.6%)
Dominant side		
Right		100 (80.6%)
Left		24 (19.4%)
Affected side		
Dominant:		61 (49.2%)
Non-dominant:		63 (50.8%)
Comorbidities:		
Cardiopathy		10 (8.0%)
Hypertension		21 (16.9%)
Hypothyroidism		12 (9.6%)
Diabetes		36 (29%)
Duration of the disease		
≤ 3 Months		39 (31.5%)
> 3 Months		85 (68.5%)
Range of motion, *degrees*		
Extension	37.1 ± 10.3	
Abduction	59.9 ± 17.7	
Internal rotation	36.4 ± 11.2	
External rotation at 90° of abduction	41.3 ± 18.9	
External rotation at 0° of abduction	28.7 ± 14.2	
Pain duration, *days*	217.7 ± 231.3	
SPADI Pain Score	57.0 ± 22.2	
SPADI Disability Score	50.2 ± 22.6	
NPRS	6.9 ± 2.0	
DASH	38.7 ± 18.9	
SF-36		
Physical Activity	76.1 ± 16.6	
Physical Role Functioning	41.3 ± 37.2	
Bodily Pain	38.7 ± 19.2	
General Health Perceptions	66.8 ± 19.7	
Vitality	53.8 ± 18.5	
Social Role Functioning	62.2 ± 26.3	
Emotional Role Functioning	59.4 ± 42.0	
Mental Health	67.0 ± 18.5	

*SPADI* Shoulder disability and pain Index, *NPRS* Numeric pain rating scale, *DASH* Disability of the arm shoulder and hand, *SF-36* Short form health survey questionnaire

### Structural validity

Considering the results of the CFA applied on the two SPADI subscales (Pain and Disability), good values were found for all indicators. The CFA results revealed a two-factor structure (CFI = 0.999; TLI = 0.997; RMSEA = 0.030; SRMR = 0.051).

### Internal consistency

Internal consistency findings are reported in Table 2. Remarkably good internal consistency was shown for both subscales (α for pain = 0.859 and α for disability = 0.895). Similarly, good results were obtained with the analysis of the item-to-total correlations: values were > 0.40 in both Pain and in Disability subscales. Alpha did not increase (it should be < α) after deleting most of the elements in both subscales, except item #9 of the Disability subscale in accordance to Cronbach’s alpha-if-item-deleted data.

**Table 2:** Item descriptive statistics and internal consistency results (*N* = 124)

*N*	*Item*	*Mean* ± *SD*	*Item-to-total-correlation (95% CI)*	*Cronbach's Alpha if item deleted*
*Pain subscale (α* = *0.859)*				
***1***	At its worst	6.8 ± 2.1	0.604 (0.479; 0.705)	0.850
***2***	Lying on the involved side	5.7 ± 2.7	0.679 (0.571; 0.764)	0.828
***3***	Reaching for something high	6.6 ± 2.7	0.649 (0.534; 0.741)	0.836
***4***	Touching the back of the neck	4.5 ± 3.0	0.704 (0.603; 0.783)	0.822
***5***	Pushing with the involved arm	5.1 ± 3.1	0.768 (0.684; 0.832)	0.804
*Disability subscale (α* = *0.895)*				
***6***	Washing the hair	4.3 ± 3.1	0.798 (0.724; 0.854)	0.869
***7***	*Washing the back*	7.4 ± 2.4	0.687 (0.581; 0.770)	0.882
***8***	Putting on an undershirt or jumper	6.0 ± 2.7	0.709 (0.609; 0.787)	0.879
***9***	Putting on a shirt that buttons down the front	3.4 ± 3.2	0.457 (0.305; 0.586)	**0.902**
***10***	Putting on pants	2.8 ± 3.0	0.748 (0.659; 0.817)	0.874
***11***	Placing an object on a high shelf	6.7 ± 2.8	0.721 (0.624; 0.796)	0.877
***12***	Carrying a heavy object	4.5 ± 3.3	0.650 (0.535; 0.741)	0.884
***13***	Removing something from your back pocket	5.1 ± 3.4	0.684 (0.577; 0.768)	0.881

Statistics values beyond the recommended cut-off are in bold

*α* *Cronbach’s alpha, CI* Confidence interval, *N/A* Not appropriate, *N* Number, *SD* Standard deviation

### Reliability

The results on reliability are shown in Table 3. Test–retest reliability (studied on 29 subjects) showed an ICC_2,1_ (95% CI) of 0.989 (0.975–0.995) for the Pain subscale and 0.990 (0.988–0.998) for the Disability subscale.

**Table 3:** Results of the Test–retest reliability and the measurement error (*N* = 29)

Scale	Test–retest reliability	Measurement error	
	ICC_2,1_ (95% CI)	SEM	MDC
**Pain** **subscale**	0.989 (0.975–0.995)	2.27	6.27
**Disability subscale**	0.990 (0.988–0.998)	2.32	6.25

*CI* Confidence interval, *ICC* Intraclass coefficient correlation, *MDC* Minimal detectable change, *SEM* Standard error of the measurement, *%* Percentage

### Construct Validity

The correlation coefficients of the *a-priori* hypotheses between SPADI subscales and the other scales administered to patients are reported in Table 4.

**Table 4:** Hypotheses testing for Spearman’s rank correlations between the Italian version of the Shoulder Pain and Disability Index (SPADI) and comparator instruments (*N* = 124)

Hypothesis testing	Estimated Correlation	Hypothesis met?
*Pain subscale*		
1.The correlation between the Pain subscale and the NPRS scores is > 0.60	0.65**	Yes
2.The correlation between the Pain subscale and the DASH total scores is > 0.60	0.62**	Yes
3.The correlation between the Pain subscale and the physical functioning (PF) subscale of the SF-36 scores is > 0.30	-0.32**	Yes
4.The correlation between the Pain subscale and the bodily pain (BP) subscale of the SF-36 scores is > 0.30 and < 0.60	-0.37*	Yes
*Disability subscale*		
1.The correlation between the Disability subscale and the DASH total scores is > 0.60	0.77**	Yes
2.The correlation between the Disability subscale and the NPRS scores is between > 0.30 and < 0.60	0.49**	Yes
3.The correlation between the Disability subscale and the role physical (RP) subscale of the SF-36 scores is > 0.30	-0.40**	Yes
4.The correlation between the Disability subscale and the social functioning (SF) subscale of the SF-36 scores is > 0.30	-0.26**	No

*SPADI* Shoulder pain and disability index, *DASH* Disability of the arm shoulder and hand, *SF-36-I* Short form health survey questionnaire, *NPRS* Numeric pain rating scale

^**^ *p* < 0,01

^*^ *p* < 0,05

The construct validity for each subscale was satisfactory. Four out of four (100%) for the Pain subscale and three out of four for the Disability subscale (75%) of the *a-priori* hypotheses were satisfied.

## Discussion

The present study suggests that SPADI can be used to assess pain and disability in patients with FS [9], given the fact that these constructs are of high concern to patients with FS [9]. Indeed, an analysis of 124 patients with idiopathic FS indicated good reliability and validity of both SPADI subscales.

Confirmatory factor analysis in our data revealed that SPADI has two dimensions (i.e., ‘‘pain’’, ‘‘disability’’). The CFA showed well-fitting values with a CFI of 0.999, a TLI of 0.997, and a low error value with an RMSEA of 0.030 and an SRMR of 0.051. The two-dimensions of the SPADI are consistent with findings from the original version of the questionnaire [7], as well as the Danish version [52], the Spanish version [47, 53], the Chinese version [51], the Greek version [54]and the other Italian version [20]. However, different findings were obtained in the Turkish [55] and Dutch [56] versions, with three factors and one-factor structures, respectively; these dissimilarities could be explained by different clinical characteristics, the shoulder pain caused by a variety of conditions (i.e. results were not specific to FS), and cultural and social factors in the patient populations studied.

The internal consistency of the SPADI was satisfactory in the Pain (*α* = *0.859*) and Disability (*α* = *0.895*) subscales. These values are consistent with those found in other studies including the development of the SPADI (in English) [7], a Chinese version validated on patients with shoulder pain [51], in a Spanish version validated on patients with six different shoulder disorders [47], and in similar Italian studies on patients with shoulder pain after neck dissection [18] or surgery for anterior instability [19].

The strong agreement between the internal consistency values for Pain and Disability subscales found in this study and those of the original version demonstrates the effectiveness of this questionnaire in assessing FS. Despite the internal consistency of each subscale, the α if-item-is-deleted of item #9 from the Disability subscale indicates a discordant value and reveals the redundancy of the item in relation to the consistency of the entire questionnaire [41]. This redundancy could be explained by the fact that item #9 investigates an activity (wearing a shirt that buttons at the front) that requires scapulothoracic abduction and internal rotation movements together with the humeroulnar flexion and extension movements. In patients with FS, glenohumeral internal rotation limitations are typically less debilitating since scapulothoracic movement helps the patient to compensate [3]. Moreover, to our best knowledge, in the available literature, there are no similar results regarding item #9 of the Disability subscale. On the other hand, the item with the highest value of α if-item-is-deleted is #6 (washing the hair*; α* = *0.902)*: this action fully involves shoulder abduction and external rotation and is one of the most provocative action in a patient with FS [6].

The construct validity of each subscale was remarkable, with 4/4 (100%) hypotheses satisfied for the Pain subscale and 3/4 (75%) hypotheses satisfied for the Disability subscale. The analysis of the construct validity brings to light knowledge similar to those that can be found in the literature [19, 47, 49, 51]; in fact, while the correlation hypotheses with NPRS and DASH were satisfied, those relating to SF-36 were not always satisfied and showed lower r_s_ values. Weaker relationships between SPADI and SF-36 was also observed in another Italian validation study [20] and could be interpreted as using the SPADI alone does not adequately evaluate the impact that FS has on a patient's quality of life. The implicit limit of this index is that it is not fully capable of assessing this condition due to its purported constructs, which minimally evaluates an aspect like social function, a domain that is not necessarily reflected in shoulder disabilities. Moreover, it could be argued that the continuous activity of nociceptive impulses occurring at the first manifestations of FS could lead to peripheral and later long-lasting central sensitization, together with an altered perception of the patient's state of health [57]. However, to date the involvement of central mechanisms in FS and the efficacy of a treatment approach focused on the central nervous system are still under study [57–62]. Consequently, the two PROMs should be administered together to more comprehensively assess the patient’s experience.

The standard deviation for the pain duration presented in Table 1 is quite large; this means that patients with different symptoms have composed the sample taken. Those data support the results of this study, demonstrating the generalizability of the findings to any patient with FS, regardless of symptoms’ entities.

According to COSMIN, a necessary condition for the use of the total score in clinical practice is that the total score is unidimensional [33, 46]. Moreover, the COSMIN recommendations [33] suggest that if the measurement instrument has two (or more) factors, the psychometric properties for these two (or more) factors should be studied separately, without considering the total score. Our results show that the SPADI has two-factors: pain and disability; therefore, the psychometric properties were examined separately for these two factors and the total score (which is affected by error) was not included in the analysis. Several cases were reported in the literature in which the total score was examined, although the SPADI has been consistently shown to have two dimensions [7, 20, 51–55]. Therefore, in clinical practice and future research, separate scores for each of the two subscales should be used rather than the total score. The present study shows good reliability and validity values of the SPADI and validates it for the assessment of patients with FS. In addition, the main contribution of this study is that the results are the first that can be fully defined in the validation process of SPADI in the patients with FS. In fact, the only other study found by the authors that examined the same patient population [11] had a smaller sample (*n* = 76) and only reported test–retest reliability and responsiveness of the SPADI.

It is worth noting the following limitations of this study: the results may lack generalizability, as this study only included patients with Idiopathic FS. Therefore, the results of this study should be applied in clinical practice only to patients diagnosed with FS. Moreover, the test–retest reliability sample consisted of only 29 patients; hence, these findings should be taken with caution. Further studies should be designed to assess other psychometric properties such as responsiveness and content validity. Finally, modern psychometric analyses (i.e., Rasch analysis) were not used to further investigate psychometric properties such as structural validity.

## Conclusion

In conclusion, SPADI has good internal consistency, reliability, and validity in a sample of patients with FS. Future studies should confirm responsiveness, interpretability, and content validity, as these properties have not been studied.

## Data Availability

The datasets used and analyzed during the current study are available from the corresponding author on reasonable request.
